# Design of an automated cell batch microinjection system based on magnetic tweezers for zebrafish embryos

**DOI:** 10.1038/s41378-026-01230-3

**Published:** 2026-03-31

**Authors:** Xiangyu Guo, Fanghao Wang, Antian Zhao, Youchao Zhang, Huanyu Jiang, Alois Knoll, Meixiao Shen, Fan Lv, Mingchuan Zhou

**Affiliations:** 1 Eye Research Center, Hangzhou Institute of Medicine, Chinese Academy of Sciences, Eye Hospital, Wenzhou Medical University, Hangzhou, China; 2 Robotic Micro-nano Manipulation Lab, College of Biosystems Engineering and Food Science, Zhejiang University, Hangzhou, China; 3 National Clinical Research Center for Ocular Diseases, Eye Hospital, Wenzhou Medical University, Wenzhou, China; 4 School of Computation, Information and Technology Technical University of Munich, Munich, Germany

**Keywords:** Electrical and electronic engineering, Optical sensors

## Abstract

Batch microinjection significantly enhances throughput and reproducibility in gene delivery and developmental studies, thereby accelerating the advancement of intelligent experimentation in the life sciences. In this work, we propose a novel visual-guided automated batch microinjection system based on magnetic tweezers, designed for zebrafish embryos. The system enables rapid and precise cell reorientation and puncture by integrating a microfluidic chip with coupled fluidic and magnetic actuation for cell manipulation. To address the challenge of robust perception in a narrow microscopic field, we introduce a microscopic manipulation perception network (MMPN), which incorporates a dual-backbone architecture and an attention mechanism to enhance feature extraction and recognition accuracy. Experimental validation demonstrates a detection mean average precision (mAP) of 98.8% and a segmentation accuracy of 98.4%. The proposed system achieves an average operation time of 33.8 seconds per cell, with a cell survival rate of 88% and a reorientation error as low as 2.1^∘^. Furthermore, successful fluorescent protein expression in zebrafish larvae confirms the effectiveness of the gene transfer. These results highlight the potential to substantially improve efficiency and reproducibility compared to manual injection. Future work will focus on extending its applicability to a broader range of cell types and enabling long-term biological studies.

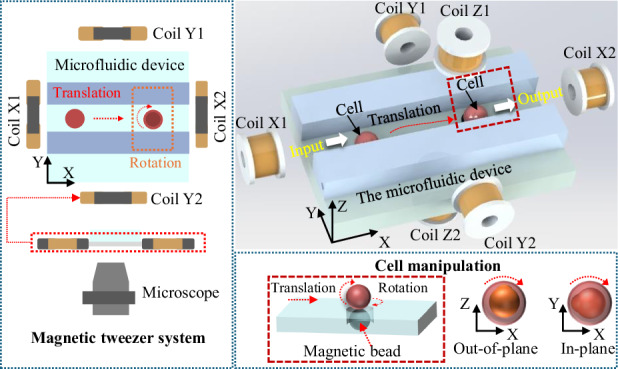

## Introduction

Zebrafish (*Danio rerio*) have emerged as the most important vertebrate model organism for developmental biology, drug discovery, and toxicology, due to their high fertilization, optical transparency, and genetic tractability^1–3^. These unique biological advantages make zebrafish particularly suitable for high-throughput experimental studies, thereby creating an urgent demand for scalable and automated methodologies, especially for genetic and chemical manipulations^4–6^. Consequently, the manual microinjection technique—a fundamental process for delivering exogenous materials into living cells^6,7^—becomes a critical bottleneck. Although widely used, conventional microinjection remains highly dependent on operator skill and is notoriously labor-intensive, error-prone, and low-throughput^8–10^. Moreover, the consistency of the results from cell microinjection is not satisfactory^11–13^, and the manual method is only suitable for handling a small number of cells. This limitation becomes more severe with the rapid growth of single-cell biomedicine, which requires precise, high-throughput, and minimally invasive manipulation technologies. Therefore, the development of an automated, high-precision microinjection system is imperative for advancing reproducible and scalable research in modern cell biology.

A critical requirement for this automated system is the ability to control cell orientation. In cell manipulation, the cell posture must be adjusted to a specific position to prevent subsequent punctures from damaging important organelles within the cells. Wang et al.^9^ employed a dual-pipette visual servo system to manipulate mouse oocytes. However, the extrusion deformation of mouse oocytes was relatively significant and easily caused the shedding of the polar body of mouse oocytes and hindered cell development. Therefore, Dai et al.^14^ proposed a minimization model to control the cell’s operating force, which reduces cell deformation to a certain extent. Chow et al.^15^ proposed a liquid metal-based electrotweezer that creates a 3D dielectrophoretic trap and 1D electrorotation. Although this method enables non-contact manipulation, the electric field could potentially damage the cell’s viability. Optical tweezers have also been used to manipulate cell posture^16,17^; however, the size of the objects that can be manipulated is limited. Additionally, high-energy laser beams can also cause cell damage^18^. Feng et al.^19^ employed an acoustic levitation method to suspend magnetic microrobots above a glass substrate and manipulated a pair of microrobots to achieve 3D posture adjustment of cells.

Researchers have attempted to improve efficiency through the manipulation of throughput. Existing systems based on robotic arms can perform cell injection but often require frequent switching between high- and low-magnification objectives, reducing operational efficiency. Meanwhile, the disordered distribution of cells in the stage increases the difficulty of localization, especially when the sample size is large. The extension of localization time further limits the overall efficiency. So, Wang et al.^20^ developed a nuclear transfer system with four microneedles for delivery, aspiration, puncture, and collection. However, due to the microscale effect of the fluid, it is difficult to ensure that only one cell is output at a time. On this basis, Liu et al.^21^ developed a batch cell manipulation system based on a microfluidic groove, which moves the carrier platform to realize the sequential injection of individual cells. However, the cells need to be preloaded into the microfluidic groove, which increases the overall operation time of the task. Guo et al.^6^ first developed a fully automatic microinjection system for zebrafish embryos integrating a closed microfluidic chip and a micro-force injector. Based on deep learning, they achieved subcellular precise positioning (with a detection accuracy of ±3 μm for the yolk center), successfully realizing continuous cell transportation, holding, injection, and real-time mechanical determination of puncture. Experiments have confirmed that the system has a batch processing capacity of 20 s per cell. However, this system lacks an active reorientation mechanism, making it difficult to precisely adjust the 3D posture of the embryos. During the injection process, it is prone to cause puncture damage to key cellular organelles due to the uncontrollable embryonic polarity direction, thereby affecting the subsequent development of the embryos. Therefore, robust visual perception is equally critical for automated microinjection.

**Fig. 1:**
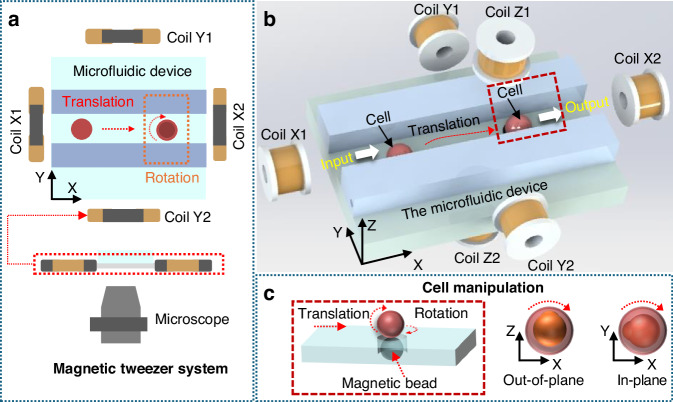
System setup. **a** Two-dimensional magnetic tweezers system based on a microfluidic device under a microscope. **b** Three-dimensional magnetic tweezers system, including four magnetic coils in the X-Y plane and two vertical coils in the Z direction. Cell transportation and reorientation. **c** Schematic diagram of cell rotation outside the plane and rotation within the plane. In (**b**, **c**), the red box region corresponds to the area where the magnetic beads are located directly beneath the cell

In response to the above problems, we present a novel method for automated batch micromanipulation of cells to enhance operational efficiency and quality Fig. 1. Magnetic tweezers are integrated into the sophisticated microfluidic chip. The fluid and magnetic field drives achieve rapid redirection and injection of cells, improving the survival rate of cells. The proposed method greatly reduces the workload of the experimenters and shortens the cycle of related research. The features and contributions of this work are listed as follows:

- A multifunctional microfluidic chip is designed with a compact structure, and a cell pose adjustment strategy is proposed based on the combination of fluid drive and magnetic field drive. Cell transportation, reorientation, injection, and collection can be completed without frequent adjustment of the objective lens, which has improved operational efficiency and prevented excessive cell deformation.
- A microscopic manipulation perception network (MMPN) is proposed to achieve robust visual perception in a narrow microscopic field, which adopts a dual backbone network combined with an attention mechanism to enhance feature extraction. The shared low-level features can achieve efficient multitask learning in a single forward propagation. The experimental results show that the detection mAP is 98.8% and the segmentation accuracy 98.4%.
- Experimental results demonstrated a single-cell operation time of 33.80 s, which is 2.3 times faster than the manual efficiency, and the reorientation error is 2.1^∘^. The puncture success rate is 100%, and the cell survival rate is 88%. The fluorescence signals of the hatching zebrafish larvae are observed clearly under the fluorescence microscope, which proves the stability and usability of this method in biological experiments.

The remainder of the article is organized as follows. First, the experimental results are introduced and discussed. Then, a conclusion summarizes the paper and presents future work. Finally, the design and manufacture of the automatic microinjection system are described in detail.

## Materials and methods

### Design and development of microinjection system prototype

**Fig. 2:**
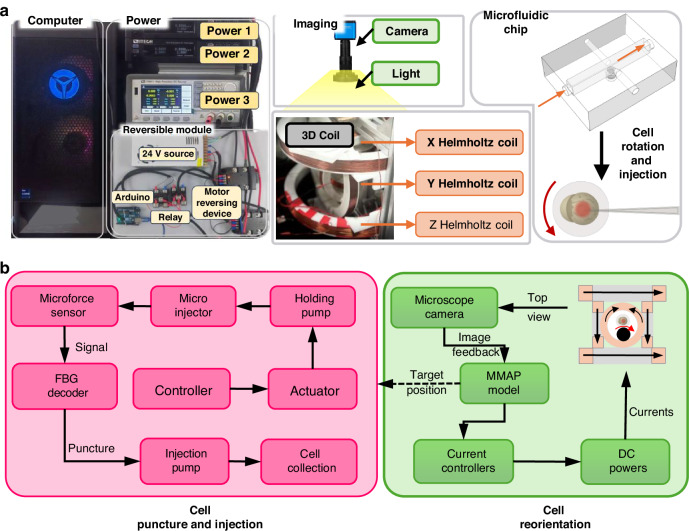
System setup of the 3D magnetic tweezers platform. **a** The system includes a computer-controlled power supply module with three independent power supply channels. A reversible motor module, based on Arduino, is powered by 24 V and uses a relay to control direction. The imaging component consists of a camera and a light source for visualization. The microfluidic chip, which guides fluid through narrow channels, is placed under the microscope. Cells are manipulated using a three-axis Helmholtz coil system, which generates a uniform magnetic field in the X, Y, and Z directions. This system can precisely rotate and position cells in the microchannel in three dimensions by applying a controlled magnetic field. **b** 3D magnetic tweezers control system diagram

The overall size of the designed chip is 30 × 20 × 10 mm^3^. It is mainly divided into the transportation area (Fig. 2a), the reorientation area, and the injection area. The transportation area includes the feeding channel and the collection channel, which are used to transport and collect cells. The chip is divided into two parts, which are constrained by alignment holes and rods to ensure that the microinjector (with FBG micro force sensors) maintains axial movement during operation, thereby preventing lateral displacement that could damage the cells. In addition, this chip is equipped with four fixed holes, which are used to firmly fix the chip on the base to ensure the stability of the operation.

The driving source of this chip includes three modes: liquid drive, gas drive, and magnetic field drive. The liquid drive utilizes the microstructures within the chip to guide the transportation of cells; the gas drive is used to fix the cells to ensure the stability of the puncture process; the magnetic field drive achieves the reorientation operation of cells through the rotation inside or outside the magnetic beads. The magnetic bead is located near the holding hole, and the visual servo system is used to precisely complete the adjustment of the cell posture.

The cell transportation module mainly consists of a micro precision guideway slide stage (CTM28-0602-50, Beijing Yixuan Technology Co., Ltd., China), a piston syringe, and a conical connecting tube. The piston syringe is fixed on the micro precision guideway slide stage, and is successively connected to the conical connecting tube and the chip dispensing channel. The magnetic control reorientation module includes a power module, three pairs of Helmholtz coils, a magnetic bead (Diameter: 1 mm), and other compatible electronic components. The inner diameters of the coil support for the *X*-axis, *Y*-axis, and *Z*-axis are 190, 140, and 180 mm, respectively, while the outer diameters are 220, 100, and 220 mm, respectively. The diameter of the copper wire is 0.8 mm. The magnetic field intensities in each axis direction are 2.08, 1.73, and 2.00 mT, respectively.

The power module consists of three DC programmable power supplies (IT-M3432, ITECH, China; IT6411, ITECH, China), providing a maximum current of 30A to the Helmholtz coils. To achieve bidirectional current control on the Helmholz coils, the power module is designed with a reversible module, which includes a 24 V power supply, an Arduino controller, two relays, and two motor reversing devices. The cell suction module is composed of a micro suction pump (CellTram® 4r Air, an air pressure micro injection instrument by Eppendorf, Germany) and a homemade micro pressure sensor. The puncture and injection module consists of a micro force injector, a micro pump (CellTram® 4r Oil, an oil pressure micro injection instrument by Eppendorf, Germany), and a micro precision guideway slide stage. The micro force injector is installed on the chip needle holder and fixed on the micro precision guideway, and is connected to the micro pump through a silicone tube. The overall control system of the machine includes an upper computer (Lenovo Yinxiao 9000K, Lenovo Group, China), a lower Arduino (UNO R3, Arduino, Italy), a driver (DM420, Beijing Yixuan Technology Co., Ltd., China), and a micro camera (Anton Star 3800, Anton Star, China). The 3D magnetic tweezers control system is shown in Fig. 2b.

### 3D magnetic tweezers and magnetic calibration

Rotational motion utilizes a constant and uniform magnetic field in space to apply torque on magnetic beads. This torque *τ* causes the magnetic beads to rotate around the direction of the external magnetic field and align uniformly. It can be described as:

1 $$\vec{\tau }=\vec{M}\times B$$

where *M* represents the net magnetic moment of the magnetic beads, and *B* represents the magnetic flux density. The rotational actuation of the magnetic bead can precisely control its torque, orientation, and angular velocity by applying appropriate excitation signals. The control equation for the magnetic field generated by the three-dimensional coil system can be expressed as:

2 $$B(t)=\left[\begin{array}{l}{B}_{x}(t)\\ {B}_{y}(t)\\ {B}_{z}(t)\end{array}\right]=\left[\begin{array}{l}A\cos (\alpha (t))\cos (\theta (t))\\ A\cos (\alpha (t))\sin (\theta (t))\\ A\sin (\alpha (t))\end{array}\right]$$

where, *B*(*t*) represents the magnetic flux density vector, with *B*_*x*_(*t*), *B*_*y*_(*t*), and *B*_*z*_(*t*) being its three-dimensional components along the *x*, *y*, and *z* axes, respectively. *A* is the amplitude (20 mT), *θ*(*t*) is the heading angle in the XY-plane relative to the *x*-axis, and *α*(*t*) is the angle relative to the XY-plane along the *z*-axis. Both *θ*(*t*) and *α*(*t*) are variables that change over time at specific frequencies. Based on the variation in *B*(*t*), the unique motion pattern of the magnetic beads and their orientation in space can be determined.

The magnetic bead is driven to rotate under an applied magnetic torque. The rotational dynamics of the bead are governed by:

3 $${\tau }_{m}-{f}_{mc}{r}_{m}-{f}_{mp}{r}_{m}={I}_{m}{\alpha }_{m}$$

where *I*_*m*_ is the moment of inertia of the magnetic bead, *α*_*m*_ is its angular acceleration, *r*_*m*_ is the bead radius, *f*_*mc*_ denotes the frictional force between the bead and the cell, and *f*_*mp*_ is the frictional force between the bead and the chip. Since no relative sliding occurs between the magnetic bead and the cell, interfacial friction enables the cell to follow the bead’s rotation. As a result, the bead transmits its angular motion to the cell via friction.

Assuming that the magnetic bead and the cell rotate synchronously with angular velocities *ω*_*m*_ and *ω*_*c*_, and satisfy the no-slip condition, we have:

4 $${\alpha }_{m}=\frac{d{\omega }_{m}}{dt}$$

For the cell, the torque generated by the frictional force *f*_*cp*_ between the cell and the chip can be expressed as:

5 $${\tau }_{c}={f}_{cp}{r}_{c}={I}_{c}{\alpha }_{c}={I}_{c}\frac{d{\omega }_{c}}{dt}={I}_{c}\cdot \frac{{\tau }_{m}-{f}_{mc}{r}_{m}-{f}_{mp}{r}_{m}}{{I}_{m}}$$

where *r*_*c*_ is the radius of the cell, *I*_*c*_ is its moment of inertia, and *f*_*cp*_ is the frictional force between the cell and the chip surface. This coupling mechanism ensures that the applied magnetic torque results in coordinated rotational motion of both the magnetic bead and the attached cell.

### Microscopic manipulation perception network

**Fig. 3:**
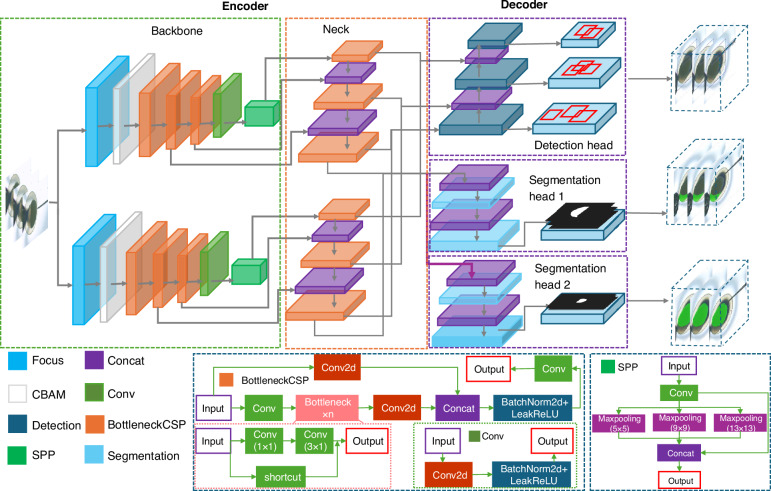
The structure of the MMPN algorithm. A novel MMPN network is proposed to achieve cell and micro-needle detection as well as cell polar segmentation. This network consists of a CSPDarkNet encoder and a dual decoder architecture, integrating residual structures, CBAM attention mechanism, and SPP modules to enhance feature extraction and context understanding. FPN realizes multi-scale feature fusion, with the detection branch locating cells and needle tips, and the segmentation branch outputting cell polar masks, featuring high accuracy and fast inference capabilities

To meet the demands of multitask perception, a novel MMPN network is proposed to detect cells and microneedles (Fig. 3), as well as segment cell polars, providing visual guidance for subsequent manipulation tasks. The network consists of one encoder and two decoders. CSPDarkNet serves as the backbone network for feature extraction, where one part undergoes a series of convolutional operations, while the other part is directly connected. Finally, the two parts are concatenated along the channel dimension. This design not only preserves the original features but also enhances the learning capacity of the network. The backbone network also includes residual blocks, which utilize skip connections from ResNet. The design of the residual blocks helps alleviate the problem of vanishing gradients in deep networks while also improving information flow, making the training of deep networks more stable. In addition, the Focus layer is incorporated, which performs efficient slicing and compression of the input. In feature extraction, the convolutional block attention module (CBAM) is used. The channel attention mechanism weights the feature maps along the channel dimension, highlighting the more distinctive feature channels in the network. It enhances the network’s ability to capture critical information and improve the quality of features.

Feature Pyramid Network (FPN) is used as the neck network, which facilitates the transmission and fusion of features in a top-down manner. This allows the network to better capture semantic information across feature maps of different resolutions. Feature maps from higher layers have lower resolution but contain stronger semantic information, while feature maps from lower layers have higher resolution but weaker semantic information. Through top-down transmission, the semantic information from higher layers is passed down to the lower layers, and fusion occurs between them. In FPN, each feature map at a given layer is fused with the high-semantic feature map from the previous layer via cross-layer connections. This fusion method ensures that the lower-level feature maps not only contain rich detail information but also retain high-level semantic information. The fusion process for each layer is typically achieved through upsampling and convolution operations. The high-level feature maps are upsampled to match the resolution of the lower-level feature maps and then fused via pixel-wise addition or convolution operations. In addition, the introduction of an Spatial Pyramid Pooling (SPP) layer between the backbone and the neck network further enhances the network’s ability to capture global context information. The SPP layer is added at the end of the backbone, which enables the network to better capture global context and effectively localize and detect multi-scale objects.

The decoder consists of the detection head and the segmentation head. The detection head is used for the position of the cell and needle tip, and outputs detection results with bounding boxes; the segmentation head is used for the segmentation of the animal pole and plant pole of cells, and outputs segmentation masks. To adapt to different-scale feature maps for needle detection, nine prior anchor points with different aspect ratios were designed, and the anchor boxes were determined using the k-means clustering algorithm. In addition, the study selected nine clustering centers and designed three different scales for each grid cell. For different levels of feature maps, the network uses scale constants to generate bounding-box priors, covering all regions from small to large. The FPN bottom-level feature map is input to the segmentation branch, and the size of the feature map is (W/8, H/8, 256). After three upsampling processes in the segmentation branch, the feature map outputs a size of (W, H, 2), representing the probability that each pixel in the input image belongs to the animal pole, plant pole, or background. Since the SPP module has been shared in the Neck network, this method does not add an SPP module in the segmentation branch like other methods, as doing so would not improve the network performance. In the upsample layer, the nearest neighbor interpolation method is used to reduce the computational cost, instead of using deconvolution. Therefore, the segmentation decoder can not only obtain high-precision outputs, but also is very fast during inference.

### Loss function and training strategy

The loss function of the designed model consists of two parts: detection loss *L*_*det*_ and segmentation loss *L*_*seg*_, which can be expressed as:

6 $${L}_{total}={\gamma }_{1}{L}_{det}+{\gamma }_{2}{L}_{seg}$$

7 $${L}_{det}={\alpha }_{1}{L}_{class}+{\alpha }_{2}{L}_{obj}+{\alpha }_{3}{L}_{box}$$

where *L*_*class*_ and *L*_*obj*_ use focal loss, with the values of parameters *α*_1_, *α*_2_, and *α*_3_ being 0.5, 0.25, and 0.25, respectively. The formula can be expressed as:

8 $${p}_{t}=\left\{\begin{array}{l}p,\,\,\,\,\,{\mathrm{if}}\,y=1\\ 1-p,\,{\mathrm{otherwise}}\end{array}\right.$$

9 $$FL({p}_{t})=-{\alpha }_{t}{(1-{p}_{t})}^{\gamma }log({p}_{t})$$

The label *y* ∈ {±1} denotes foreground and background. The model predicts a probability *p* ∈ [0, 1] of being foreground. To handle class imbalance and sample difficulty, the focal loss introduces a category weight *α*_*t*_ and a modulation factor ${(1-{p}_{t})}^{\gamma }$, where *γ* adjusts the focus on hard examples. When *p*_*t*_ is near 1 (i.e., correct prediction), the loss contribution becomes negligible, reducing focus on easy samples. This balances training by emphasizing misclassified or minority samples. In this work, *α*_*t*_ = 0.25, *γ* = 2. *L*_*seg*_ is calculated as:

10 $${L}_{seg}={L}_{Tversky}+{L}_{Focal}$$

where *L*_*Tversky*_ is the Tversky loss, and *L*_*Focal*_ is the focus loss.

The study designed a three-stage training strategy (Table 1), which reduced the complexity of the parameter space and improved the convergence speed. In Stage I, the segmentation head is frozen, and only the encoder and detection head are trained to enhance object localization and classification. In Stage II, the encoder is frozen, while both the detection and segmentation heads are trained. This addresses the mismatch between the randomly initialized segmentation head and the encoder features learned in Stage I, promoting better feature alignment. In Stage III, all network parameters are unfrozen for end-to-end joint training, enabling deep feature fusion and synergy between detection and segmentation tasks. This progressive unfreezing strategy improves model accuracy and robustness through phased adaptation and collaborative optimization.

**Table 1:** Algorithm 1: training strategy

Algorithm 1:	Training strategy
**Input**	Neural network *F* with parameters *Θ* = {*θ*_*enc*_, *θ*_*det*_, *θ*_*seg*_}
	Training set *T*; convergence threshold *thr*; loss function *L*_*total*_
**Output**	Trained network *F*(*X*, *Θ*)
1	Procedure: **TRAIN**(*F*, *T*)
2	Repeat
3	Sample mini-batch (*x*_*s*_, *y*_*s*_) from *T*
4	*L* ← *L*_*total*_(*F*(*x*_*s*_, *Θ*), *y*_*s*_)
5	$\Theta \leftarrow \arg \mathop{\min }\limits_{\Theta }L$
6	Until *L* < *thr*
7	End
8	*Θ* ← *Θ*⧹{*θ*_*seg*_} // freeze segmentation head
9	**TRAIN**(*F*, *T*)
10	*Θ* ← (*Θ* ∪ {*θ*_*seg*_})⧹{*θ*_*enc*_, *θ*_*det*_}
	// freeze encoder and detection head, activate segmentation head
11	**TRAIN**(*F*, *T*)
12	*Θ* ← *Θ* ∪ {*θ*_*seg*_, *θ*_*det*_}
	// unfreeze all parameters
13	**TRAIN**(*F*, *T*)
14	Return *F*(*X*, *Θ*)

### Evaluation metrics

The performance of the proposed algorithm is evaluated using standard metrics. Accuracy (Acc) measures overall classification correctness, defined as the ratio of correctly predicted samples to the total number.

11 $$Acc=\frac{TP+TN}{TP+FP+TN+FN}$$

where *TP*, *TN*, *FP*, and *FN* represent true positives, true negatives, false positives, and false negatives, respectively.

Recall (*R*) measures the proportion of true positives identified among all actual positives, reflecting the model’s ability to detect positive samples.

12 $$R=\frac{TP}{TP+FN}$$

Average precision (AP) is employed to evaluate detection quality across varying thresholds. It is computed as the area under the precision-recall curve, reflecting the balance between precision and recall.

13 $$AP={\int }_{0}^{1}P(R)dR$$

Mean average precision (mAP) provides an overall evaluation by averaging the AP values across *N* object classes.

14 $$mAP=\frac{1}{N}\mathop{\sum }\limits_{i=1}^{N}{\mathrm{AP}}({N}_{i})$$

In particular, mAP_0.5_ denotes mAP at an IoU threshold of 0.50, while mAP_0.5:0.95_ represents the average mAP across IoU thresholds from 0.50 to 0.95 in increments of 0.05.

Intersection over union (IoU) quantifies the overlap between predicted and ground truth bounding boxes and is used to assess localization precision.

15 $$IoU=\frac{{A}_{o}}{{A}_{u}}$$

where *A*_*o*_ is the intersection area and *A*_*u*_ is the union area.

For multi-class segmentation tasks, mean intersection over union (mIoU) represents the average IoU across all *k* categories.

16 $$mIoU=\frac{1}{k}\mathop{\sum }\limits_{i=1}^{k}\frac{Q{\cap }G}{Q{\cup }G}$$

where *Q* and *G* denote the predicted and ground truth regions, respectively.

Frames per second (*FPS*) is used to evaluate runtime efficiency by measuring the number of image frames processed per second, where a higher FPS indicates better real-time performance.

17 $$FPS=\frac{1}{{T}_{{\rm{f}}{\rm{r}}{\rm{a}}{\rm{m}}{\rm{e}}}}$$

where *T*_frame_ is the time required to process a single frame.

The performance of the micro-injection system is evaluated as the following metrics. Operation efficiency *η*:

18 $$\eta =\frac{{T}_{{\mathrm{tim}}}}{{N}_{{\mathrm{tot}}}}$$

where ${T}_{{\mathrm{tim}}}$ is the total time required to successfully inject *N*_puc_ cells, and *N*_tot_ is the total number of cells.

Puncture success rate Φ:

19 $$\Phi =\frac{{N}_{{\mathrm{puc}}}}{{N}_{{\mathrm{tot}}}}\times 100 \%$$

where *N*_puc_ is the number of successfully punctured cells. Cell reorientation error *θ*_*e*_:

20 $${\theta }_{e}=| {\theta }_{r}-{\theta }_{t}|$$

where *θ*_*r*_ is the desired rotation angle, and *θ*_*t*_ is the angle at time *t*.

Cell survival rate *N*:

21 $$N=\frac{{N}_{{\mathrm{sur}}}}{{N}_{{\mathrm{puc}}}}\times 100 \%$$

where *N*_sur_ is the number of cells that are successfully punctured and eventually hatch into larvae.

## Results and discussion

### Preparation of the experimental study

One day prior to the experiment, adult male and female zebrafish are placed into breeding tanks at a male-to-female ratio of 1:2, separated by a transparent partition. On the day of the experiment, the partition is removed to allow mating. Following spawning, embryos are collected for experimental use. Microinjection is typically carried out at the 1-cell to 4-cell stage (diameter: 800–1000 μm). The experimental vector used is *PCS2* (vector: *tol2*-*α*actin-egfp) at a concentration of 150 ng/μL, with 2 nL injected into each embryo.

A total of 2000 original images were collected for this study. To improve the generalization ability of the model, various data augmentation techniques, including rotation, scaling, cropping, and flipping, are applied to increase the diversity of the training data. As a result, the dataset expands to 5000 images. The images are then divided into training, validation, and test sets in a 7:1:2 ratio. Annotation of object detection bounding boxes and segmentation masks is performed using the Labelme tool (v5.4.1). The annotated dataset is organized in the PASCAL VOC format for compatibility and ease of use. Specifically, the dataset is structured into four folders: Images, which contains all original images; Detection-Annotation stores the object detection annotations in .json format; and Segmentation-Animal and Segmentation-Plant, which contain segmentation masks in .jpg format for animal and plant categories, respectively. The folder structure allows efficient organization and subsequent use. To accelerate model training, the resolution of all images is standardized to 640 × 400 pixels. All training is conducted end-to-end using the PyTorch 1.11.0 framework on a single NVIDIA RTX 3060 GPU running Ubuntu 18.04.

### Performance evaluation of visual perception algorithms

**Fig. 4:**
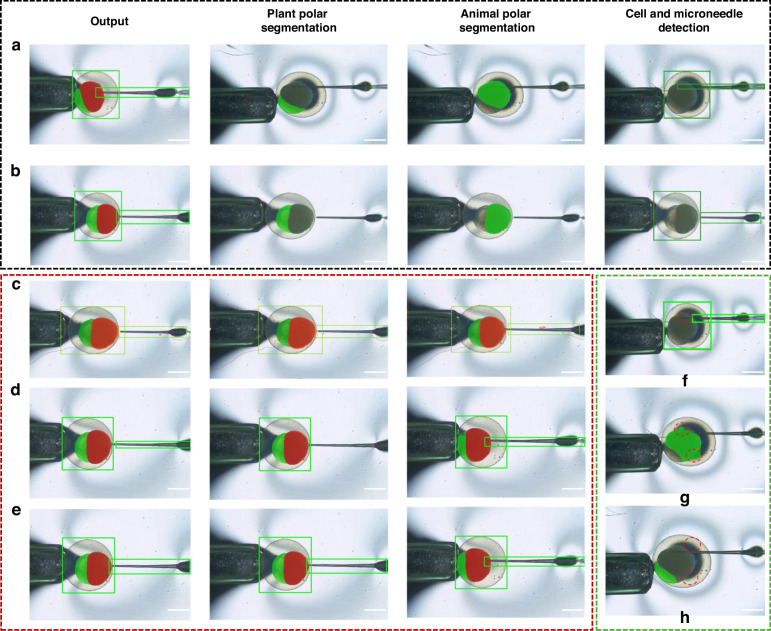
Visual perception algorithm results. **a**, **b** MMPN algorithm detects cell and microneedle (green boxes), and segments animal and plant poles (green and red masks). **c–e** show ablation experiment results. **f**–**h** present examples of incorrect detection and segmentation by other algorithms. The scale bar is 500 μm

As illustrated in the Fig. 4a, b, the proposed method achieves mAP_0.5_ of 98.8% for detection and Acc of 98.4% for segmentation. Three groups of ablation experiments are designed, each corresponding to a different variant of the MMPN model (Table 2). The first group uses a configuration consisting of one encoder and two decoders, serving as the baseline network. This baseline model achieves a *R* of 96.4%, an mAP_0.5_ of 95.4%, and an mAP_0.5:0.95_ of 51.8%. The segmentation accuracy for the animal pole and plant pole reaches 96.5%, with mIoU of 98.9%. However, the results show some issues, such as incorrect segmentation and inaccurate detection results (Fig. 4c).

**Table 2:** Results of ablation experiments on different variants of MMPN-net

Method	Recall (%)	mAP_0.5_ (%)	mAP_0.5:0.95_ (%)	Acc (%)	IoU (%)	mIoU (%)
1	96.4	95.4	51.8	96.5	95.6	98.9
2	98.6	96.8	52.4	97.3	96.2	**99.9**
3	**99.8**	**98.8**	**52.8**	**98.4**	**96.8**	**99.9**

1 = Baseline model (single encoder (single backbone network) + dual decoders)

2 = Baseline model + CBAM

3 = MMPN model (dual backbones + CBAM)

Bold values indicate the best performance for each metric

In the second group, the CBAM attention mechanism is introduced based on the baseline network. As a result, all evaluation metrics show significant improvements. *R* increases to 98.6%, mAP_0.5_ and mAP_0.5:0.95_ reach 96.8% and 52.4%, respectively. These improvements indicate that the attention mechanism effectively enhances the model’s focus on important features. The segmentation accuracy increases to 97.3%, and the mIoU remains high at 99.9%. Nevertheless, a few cases of missegmentation are still observed (Fig. 4d).

The third group of experiments employs the full MMPN model. The configuration achieves a *R* of 99.8%, with mAP_0.5_ and mAP_0.5:0.95_ improving to 98.8% and 52.8%, respectively. These improvements not only reflect the enhancement of the encoder’s feature extraction capability but also demonstrate the model’s flexibility and effectiveness in handling complex features and multi-scale information. The segmentation accuracy further improves to 98.4%, the IoU reaches 96.8%, and the mIoU remains at 99.9%, indicating superior segmentation performance (Fig. 4e). Overall, each group of experiments is significantly superior to the previous one, showing notable progress in both detection and segmentation metrics. Figure 4f–h presents the detection results of several mainstream algorithms. However, these results have certain limitations, including inaccurate objection and missegmentation. Tables 3 and 4 provide detailed information on the comparison results with different algorithms.

**Table 3:** Comparison of object detection algorithms

Method	R%	mAP(%)	FPS
YOLOX	96.9	90.2	30
YOLOP	98.6	98.2	20
MobiletNetv3	96.4	97.9	23
ResNet	97.0	98.42	25
Ours	99.8	98.8	25

**Table 4:** Comparison of object segmentation algorithms

Method	Acc%	mIoU(%)	FPS
Mask R-CNN	94.5	97.2	25
YOLOP	94.5	96.8	20
MobileNetv3	99.8	98.4	23
ResNet	97.6	99.9	25
Ours	98.4	99.9	25

### The performance analysis of cell reorientation module

**Fig. 5:**
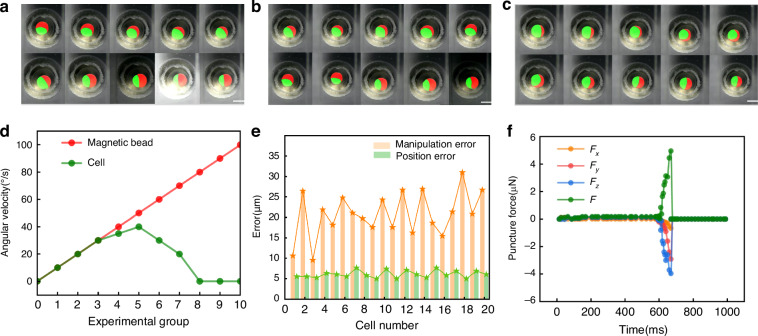
Cell manipulation. **a**–**c** Examples of in-Plane and out-of-plane cell rotation. **d** The angular velocity relationship between the magnetic bead and the cell. **e** Micro needle manipulation error and position error. **f** Puncture force of cell. The scale bar is 500 μm

To ensure that magnetic beads reliably drive cell rotation, this study designs ten groups of experiments to investigate the effect of bead angular velocity on cell behavior. An appropriate angular velocity parameter is selected to achieve stable rotation. The angular velocity of the magnetic beads starts at 0^∘^/s and increases incrementally by 10^∘^/s up to 100^∘^s. In each group, the angular velocities of the magnetic bead and the cell are recorded in real time to observe their relationship. As shown in Fig. 5a–d, at lower angular velocities (Groups 1 to 3), the bead and the cell rotate nearly synchronously, and the cell consistently follows the motion of the bead. This implies that at lower rotational speeds, the adhesive interaction between the magnetic bead and the cell provides enough resistance to counteract slippage, allowing consistent rotational manipulation. However, as the bead’s angular velocity increases (from Group 4 onward), the cell’s rotational speed gradually decreases and deviates from the bead’s speed. This deviation is attributed to the increased inertial effects of the magnetic bead at higher speeds, which intensify the relative slippage and prevent the cell from following the rotation stably. Therefore, an angular velocity of 15^∘^/s is selected to maintain stable actuation in subsequent experiments.

The performance of the cell reorientation module is evaluated in terms of success rate, operation time, angular rotation error, and failure causes, providing a reference for future optimization. A total of 20 samples are used, with each undergoing three rotation trials. At the beginning of each trial, the orientation of the cell’s polar body is randomly distributed. In the out-of-plane rotation tests, a trial is considered successful if the polar body appears predominantly within the imaging plane after rotation^22,23^. In the in-plane rotation tests, the objective is to align the polar body to the 9 o’clock position within 20 s. In the out-of-plane tests, 58 out of 60 trials are successful, with a success rate of 96.67%. The primary cause of failure is the failure to detect the polar body. In the in-plane tests, 54 out of 58 trials are successful, and the 4 failures are due to unexpected out-of-plane coupling, with a success rate of 93.10%. The average total rotation time for each sample is 13.5 s, with an out-of-plane rotation time of 6.2 s and an in-plane rotation time of 7.3 s. The visual position error of the microneedle is 5.03 μm, and the manipulation error is 21.18 μm (Fig. 5e). When the cell is punctured, the mechanical signals undergo a sudden change (Fig. 5f).

### The performance analysis of microinjection system

**Fig. 6:**
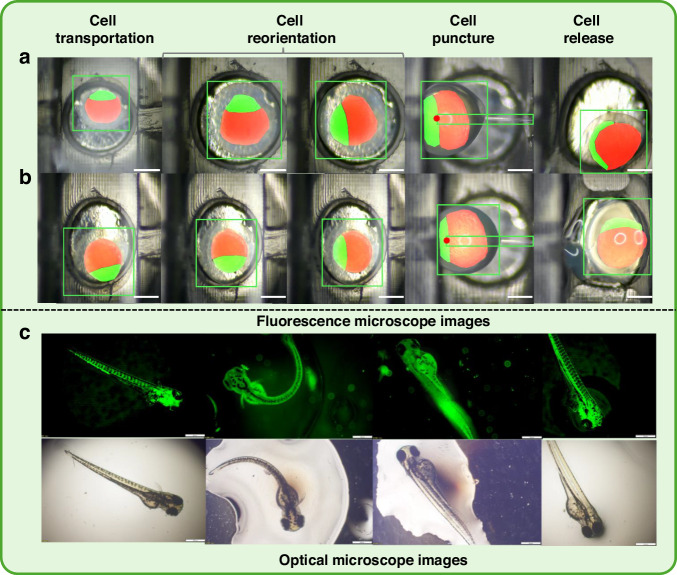
Visualization of manipulation examples. **a**, **b** display different manipulation instances, including cell transportation, reorientation, puncture, and release, with the MMPN algorithm for cell and microneedle detection and polarity segmentation. **c** Fluorescence microscopy images showing strong GFP expression in hatched zebrafish larvae, confirming that injected genetic material was successfully delivered and stably expressed. These results provide biological evidence of both precise injection and embryo viability, demonstrating the practical utility of the system. The scale bar is 500 μm

**Fig. 7:**
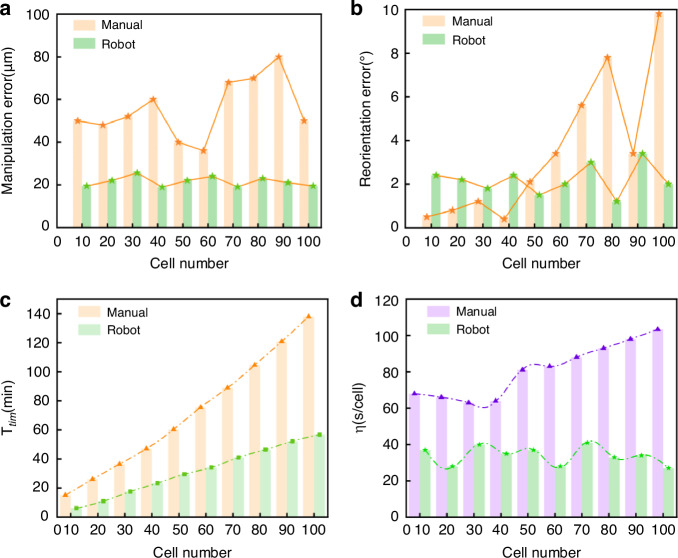
Performance comparison between manual and robotic methods. A total of 100 cells are manipulated by the manual method, while another 100 cells are manipulated by the proposed method. The experiment is divided into ten groups, with ten samples in each group. For each group, the operation error, reorientation error, operation time, and operational efficiency are calculated. **a** Manipulation error, **b** reorientation error, **c** operation time, and **d** survival rate

A total of 100 cells are manipulated by the manual method, and another 100 cells are manipulated by the proposed method. The corresponding results are shown in Table 5 and Figs. 6, 7. The results show that the manual manipulation of 100 cells takes 8042 s, averaging 80.42 s per cell. In contrast, the proposed method completes the same task within 3380 s, averaging 33.80 s per cell. It reduces the time by 46.22 s and achieves 2.3 times higher efficiency. In the manual process, it is necessary to frequently switch the magnification of the microscope to locate the cells and the micro needle, which significantly increases the time consumption. In addition, manual performance tends to degrade over time, likely due to operator fatigue. Green fluorescence is observed in the hatched larvae under a fluorescence microscope, indicating the effectiveness of the proposed method (see Fig. 6c). Green fluorescence expression confirms that the injected genes or molecules have successfully entered the cells and are expressed in the embryos, validating the success of both the cell puncture and gene transfection processes. This result not only demonstrates the precision of the proposed method in cellular operations but also provides robust technical support for future genetic research.

**Table 5:** Comparative analysis of different cell manipulation methods

Metric	Manual method	This work
*N*_tot_	100	100
*θ*_*e*_ (^∘^)	4.5	2.1
${T}_{tim}$ (s)	8042	3380
*N*_puc_	92	100
*N*_sur_	66	88
*ϕ* (%)	92	100
*R* (s/cell)	29.40	13.50
*η* (s/cell)	80.42	33.80
*N* (%)	66	88

*R* represents the average time for adjusting the posture of a single cell

For cell reorientation, the operational efficiency of the manual method is 29.40 s per cell, with a reorientation error of 4.50^∘^. The operational efficiency of the proposed method is 13.50 s per cell, with a reorientation error of 2.1^∘^. Compared with the manual method, the proposed method can effectively shorten the cell posture adjustment time and improve the operational efficiency. The reason why manual cell posture adjustment takes a long time is that it requires both hands to control one micro-manipulation arm separately and to coordinate highly to pick up and complete the task. The manipulation is difficult and very time-consuming. In contrast, this paper adopts the magnetic field driving method for adjustment, simplifying the operation process. It uses the friction between the magnetic beads and the cells to achieve in-situ rotation, avoiding the harm of manual operation to squeeze the cells.

## Discussion

This study develops a novel automated batch microinjection system based on magnetic tweezers for zebrafish embryos, which integrates a cell transportation module, a reorientation module, and a puncture and injection module to achieve continuous cell manipulation. The proposed method achieves an average manipulation time of 33.80 s per cell, with a reorientation time of 13.50 s per cell, a redirection error of 2.1^∘^, and a post-manipulation cell survival rate of 88%. Compared with the manual approach, the manipulation time per cell is reduced by 46.22 s. In conventional approaches, cells are placed within the microscopic field of view. A low magnification objective is employed to search for the target cell and microneedle, and the cells are punctured under high magnification. Frequent switching between microscope objectives restricts improvements in operational efficiency. Manual manipulation of a single cell typically requires 37.08 to 120 s, with a survival rate of 50% to 70%^21,24^, both of which are markedly inferior to the performance achieved by the automated method. These results are consistent with the findings of this work.

Table 6 summarizes the performance of different cell manipulation methods. The literature^25^ indicates that electro-osmotic flow is used for the delivery of external materials, with the injection volume controlled by voltage. However, the potential impact of the properties of the external materials on this process still requires further verification. Pressure injection does not have this risk. The results of literature^12^ indicate that 100 zebrafish embryo cells are directly injected, of which 84 successfully hatch into zebrafish, with a survival rate of 84%. And fluorescence signals are observed under the fluorescence system. Compared with the proposed method, the survival rate increases by 4%, and the manipulation time is approximately 21 s per cell. This difference may relate to the random distribution of cell posture. Without reorientation adjustment, the microneedle may directly puncture the animal pole, resulting in the death of some cells. Thanks to the proposed visual perception algorithm, cellular feature information is obtained to guide cell reorientation and microinjection manipulation. This method is highly suitable for achieving high efficiency in cell manipulation. Furthermore, the manual operation error and redirection error are 55.51 μm and 4.5^∘^, respectively. The coefficients of variation for the manual operation error and redirection error across five sets of manual operations are 25.48% and 70.92%. The coefficients of variation for the robotic operation error and redirection error are 11.19% and 23.22%. The consistency of the proposed method is higher than that of manual operation, and individual operational experience is related to the outcomes.

**Table 6:** Comparison of different cell manipulation methods

Methods	Subject	Reorientation error(^∘^)	Efficiency(%)	Success rate(%)	Survival rate(%)	
Exhalation/inhalation control^9^	Mouse oocyte	3.9	11.2	94	–	Cell deformation
Minimal cell deformation^14^	Mouse oocyte	1	10–15	95	–	Cell deformation
Fluidic control^26^	Mouse oocyte	1.9	23.6	97.6	–	–
Minimal rotational force^27^	Mouse oocyte	1.2	28.6	90	–	Cell deformation
Assembly-line style^20^	Pig oocyte	–	50	100	–	Unhatched
Microfluidics^28^	Fibroblast Cardiomyocyte	1.7	–	–	58.5-88.4	Extra 10 min
Microfluidics^25^	Zebrafish embryo	–	20	–	80	Electroosmosis
Rotational injection^29^	Zebrafish embryo	–	>17.3	–	–	Unhatched
Microfluidics^30^	Zebrafish	–	41.1	–	81.42	–
Microfluidics^31^	Zebrafish	–	18.61	–	90	–
Microfluidics^12^	Zebrafish embryo	–	20	–	84	Hatched
This work	Zebrafish embryo	**2.1**	**33.80**	–	**88**	Hatched

Bold values represent the results of our proposed method

Compared with earlier studies, the cell reorientation error of 2.1^∘^ essentially meets the operational requirements. The mechanical manipulation method based on robotic arms involves prolonged operation time and often results in unavoidable cell compression, leading to significant deformation. Although the method based on minimal rotational force mitigates this issue to some extent, it does not eliminate it fundamentally. In contrast, this study employs a magnetic field-driven approach to adjust cell orientation. By exploiting frictional forces to induce rotation, the method effectively avoids cell deformation. The success rate for out-of-plane rotation reaches 96.67%, while the in-plane rotation success rate is 93.10%.

The system is applicable to medical drug screening and biological breeding research, significantly improving research efficiency and cell survival rate, thereby demonstrating strong potential for practical applications. Its design also supports studies on model organisms such as zebrafish larvae and small animals like Caenorhabditis elegans (length: 1 mm), although the chip dimensions need to be adjusted according to different biological targets.

## Conclusion

In this study, we present a novel batch cell manipulation approach that enables precise cell reorientation and puncture, substantially improving operational efficiency and cell viability. A magnetic particle is embedded within a closed microfluidic chip, which allows the cell posture to be adjusted by varying the external magnetic field, thereby minimizing cellular deformation. To further enhance operational precision, an MMPN model is designed to obtain feature information about cells, microneedles, and intracellular organelles, which guides the robotic execution of micromanipulation tasks. Experimental results demonstrate that the average operation time for a single cell is 33.80 s, which is 2.3 times more efficient compared with the manual method. The cell reorientation error is reduced to 2.1^∘^, and the post-manipulation survival rate reaches 88%. Moreover, fluorescence microscopy revealed distinct fluorescent signals in the hatched zebrafish larvae. So, the results provide evidence for the feasibility and robustness of the developed system in biological experimentation.

## Data Availability

The data that support the findings of this study are available from the corresponding author upon reasonable request.
